# Transcriptome Analysis of *Salvia miltiorrhiza* under Drought Stress

**DOI:** 10.3390/plants13020161

**Published:** 2024-01-06

**Authors:** Siwei Zhang, Xinlan Qi, Ruiyan Zhu, Dongdong Ye, Minyu Shou, Lulu Peng, Minghua Qiu, Min Shi, Guoyin Kai

**Affiliations:** 1 Laboratory of Medicinal Plant Biotechnology, School of Pharmaceutical Sciences, Zhejiang Chinese Medical University, Hangzhou 310053, China; zsw885900@163.com (S.Z.); qixinlan1020@163.com (X.Q.); yedongdong0710@163.com (D.Y.); smy252900@126.com (M.S.); 15290280761@163.com (L.P.); 2 College of Horticulture, Shenyang Agricultural University, Shenyang 110866, China; 3 State Key Laboratory of Phytochemistry and Sustainable Utilization of Plant Resources in Western China, Kunming Institute of Botany, Chinese Academy of Sciences, Kunming 650201, China; mhchiu@mail.kib.ac.cn

**Keywords:** *Salvia miltiorrhiza*, phenolic acids, drought stress, ABA-dependent signaling pathways, transcription factors

## Abstract

Phenolic acids are one of the major secondary metabolites accumulated in *Salvia miltiorrhiza* with various pharmacological activities. Moderate drought stress can promote the accumulation of phenolic acids in *S. miltiorrhiza*, while the mechanism remains unclear. Therefore, we performed transcriptome sequencing of *S. miltiorrhiza* under drought treatment. A total of 47,169 unigenes were successfully annotated in at least one of the six major databases. Key enzyme genes involved in the phenolic acid biosynthetic pathway, including *SmPAL*, *SmC4H*, *Sm4CL*, *SmTAT*, *SmHPPR*, *SmRAS* and *SmCYP98A14*, were induced. Unigenes annotated as laccase correlated with *SmRA*S and *SmCYP98A14* were analyzed, and seven candidates that may be involved in the key step of SalB biosynthesis by RA were obtained. A total of 15 transcription factors significantly up-regulated at 2 h and 4 h potentially regulating phenolic acid biosynthesis were screened out. TRINITY_DN14213_c0_g1 (AP2/ERF) significantly transactivated the expression of *SmC4H* and *SmRAS*, suggesting its role in the regulation of phenolic acid biosynthesis. GO and KEGG enrichment analysis of differential expression genes showed that phenylpropanoid biosynthesis and plant hormone signal transduction were significantly higher. The ABA-dependent pathway is essential for resistance to drought and phenolic acid accumulation. Expression patterns in drought and ABA databases showed that four PYLs respond to both drought and ABA, and three potential SnRK2 family members were annotated and analyzed. The present study presented a comprehensive transcriptome analysis of *S. miltiorrhiza* affected by drought, which provides a rich source for understanding the molecular mechanism facing abiotic stress in *S. miltiorrhiza*.

## 1. Introduction

*Salvia miltiorrhiza* Bunge (also termed as Danshen) is a traditional Chinese medicine with a rich history for various health benefits, which is commonly referred to as “Red Sage” due to the red color [1,2]. Danshen is native to China and cultivated in other Asian countries as well. The primary bioactive constituents of *S. miltiorrhiza* include tanshinones and phenolic acids, which contribute to potential positive effects on the cardio-cerebral vascular system [3]. Danshen-based preparations are known for their cardiovascular benefits, providing support for blood vessels, heart function and other health issues. Formulations like Danshen injection and Compound Danshen Dripping Pills are developed by extracting the bioactive component phenolic acids from Danshen roots. Overharvest coupled with unsustainable collection has led to a decrease in the natural Danshen population. Therefore, there is a great necessity to increase the yield to meet the growing clinical needs.

There are several distinct components, such as rosmarinic acid (RA) and salvianolic acid B (SalB), that belong to the phenolic acid class, exhibiting a range of bioactive properties, such as antibacterial, antioxidant and antiviral. The biosynthesis pathway of phenolic acids starts with the conversion of phenylalanine to 4-coumaryl CoA, the process undergoes catalysis of phenylalanine ammonia-lyase (PAL), cinnamate 4-hydroxylase (C4H) and 4-coumarate: CoA ligase (4CL) [4]. Another precursor 3,4-hydroxyphenyllactic acid is produced under catalyzation of tyrosine aminotransferase (TAT), 4-hydroxyphenylpyruvate reductase (HPPR) and unknown CYP450 enzyme. Further modifications medicated by rosmarinic acid synthase (RAS) and cytochrome P450-dependent mono-oxygenase (CYP98A14) occur between the two intermediates to produce RA, and then RA is synthesized into SalB through unknown enzymatic reactions. It was speculated that laccase (LAC) may be the key enzyme in the conversion of RA to SalB [5], which still needs to be further studied.

In recent years, various strategies including elicitation, genetic manipulation of rate-limiting enzymes, heterologous gene overexpression, blocking of competitive branches, transcription regulation and post-translational modification have been applied to promote phenolic acid production. For instance, overexpressing single genes, such as *PAL*, *TAT*, *HPPR*, *C4H*, *RAS* or *CYP98A14*, achieved increased phenolic acid production, and co-expressing *TAT* and *HPPR* produced the highest amount of RA, which was 4.3-fold higher than that of the wild type [6,7]. Moreover, suppression of *HPPD* also led to increased phenolic acid content [6]. The introduction of exogenous genes such as *AtPAP1* in hairy roots of *S. miltiorrhiza* has also been investigated to enable enhanced phenolic acid production [8]. Exogenous application of phytohormones triggered the activation of pathway genes to induce the production of phenolic acids. For example, the application of 0.1 mmol/L of methyl jasmonate (MeJA) led to the upregulation of key genes to promote RA and lithospermic acid B (LAB) accumulation [9]. After salicylic acid (SA) treatment for 8 h, phenolic compounds accumulated in the cell suspension and stimulated the activity of PAL [10]. Abscisic acid (ABA) effectively induced the production of phenolic acids by impacting PAL and TAT activity [11]. Meanwhile, multiple transcription factors (TFs) (WRKY, MYB, AP2/ERF, bHLH, bZIP) have been characterized as important players in the regulation of phenolic acid biosynthesis. For example, overexpression of *SmPAP1* increased the accumulation of RA and SalB, directly activating *SmPAL1* and *SmC4H* [12]. JA-responsive *SmMYB1* is involved in the transcriptional control of *SmCYP98A14* to promote phenolic acid biosynthesis [13]. MeJA-induced *SmERF115* upregulated phenolic acid content with *SmRAS* as the target [14]. And bHLH TF, such as *SmMYC2*, has been shown to significantly promote the accumulation of phenolic acids [15]. ABA-responsive TF *SmbZIP1* enhanced the yield of phenolic acids by controlling the biosynthesis gene *SmC4H* [16]. The SmKFB5 protein controlled the degradation of SmPAL to affect phenolic acid biosynthesis [17].

Among the abiotic stresses that plants faced, drought showed the greatest impact on plant growth, development and metabolism. Plants synthesize ABA under drought conditions, and the ABA-dependent pathway is the core of resistance to drought stress, which may also participate in phenolic acid accumulation. In the absence of ABA, PP2C members dephosphorylate SnRK2 subfamily III members, resulting in the loss of the phosphorylation function of SnRK2 subfamily III members. When plants are exposed to drought stress, ABA biosynthesis is increased, and ABA directly interacts with PP2CA subfamily members after binding to ABA receptors PYL/PYR/RCAR to release TFs and functional genes downstream of phosphorylation of SnRK2 subfamily III members to play a role in drought resistance and secondary metabolism [18]. Drought stress impacted the accumulation of flavonoids in *Astragalus membranaceus* (Fisch.) Bunge and the content of calycosin-7-O-b-D-glycoside reached the peak after 14 days of drought treatment [19]. Moderate water deficit significantly increased the contents of total phenols, flavonoids and ascorbic acid in leaves of *Citrus aurantium* L. [20]. Short-term water scarcity increased artemisinin production, but long-term drought stress resulted in decreased levels of artemisinin [21]. With drought treatment, the expression of *PAL*, *C4H*, *4CL* and *CHS* in *Lotus corniculatus* subsp. *japonicus* (Regel) H. Ohashi was increased, which led to the accumulation of quercetine and kaempferol [22]. Medium drought stress promoted the accumulation of total phenolic acids in *S. miltiorrhiza* leaves through the regulation of pathway genes [23]. However, the specific mechanism is still unclear.

To investigate the effect of drought stress on *S. miltiorrhiza*, comprehensive transcriptome sequencing was performed to identify potential genes participating in, or transcription factors regulating, phenolic acid biosynthesis. Moreover, genes associated with drought stress were analyzed, which helps in a deeper exploration of the mechanism of *S. miltiorrhiza* in response to abiotic stress.

## 2. Results

### 2.1. Accumulation of Phenolic Acids in S. miltiorrhiza under Drought Stress

**Figure 1:**
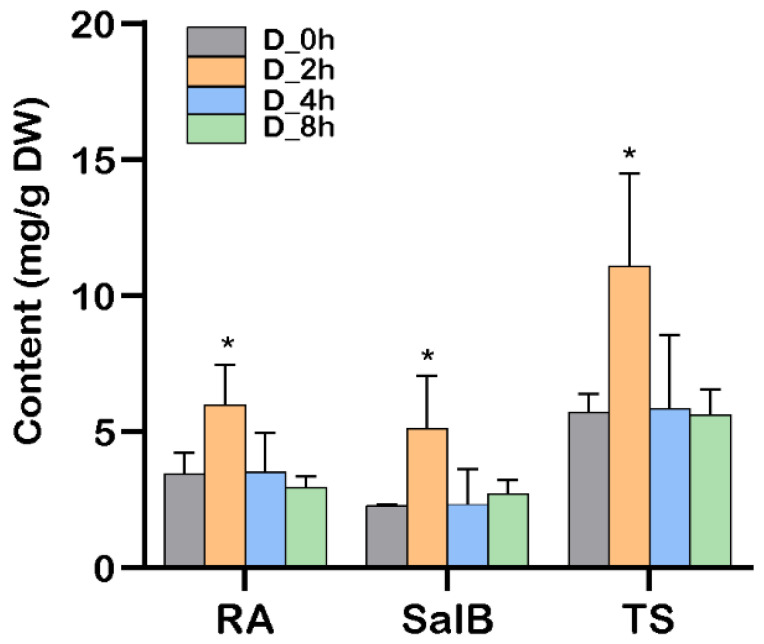
Phenolic acid content in drought-treated *S. miltiorrhiza* plants at 0, 2, 4, 8h. D_0h, drought treatment for 0h (before treatment); D_2h, drought treatment for 2h; D_4h, drought treatment for 4h; D_8h, drought treatment for 8h. RA, rosmarinic acid; Sal B, salvianolic acid B; TS, total phenolic acids. Error bars indicate SD (*n* = 3), * *p* < 0.05.

The content of RA and SalB from drought-treated plants under different time points was detected by HPLC (Figure 1). The phenolic acid content reached the maximum after 2 h of drought treatment and then declined. HPLC results revealed that moderate drought will promote phenolic acid accumulation in *S. miltiorrhiza*.

### 2.2. Transcriptome Sequencing and De Novo Assembly

In order to further study the regulation mechanism of phenolic acid accumulation, *S. miltiorrhiza* was induced with natural drought for 0, 2, 4 and 8h for transcriptome sequencing, respectively. Four cDNA libraries containing 52,876,386, 53,994,332, 50,632,334 and 53,097,080 raw reads were constructed (Table 1). The length of the longest and the shortest unigene was 13,359 and 201 bp, respectively, with an average length of 1098.34 bp and an N50 length of 1738 bp. GC content was 43.47%, and BUSCO score was C: 88.2% (Table 2).

### 2.3. Functional Annotation and Classification of Unigenes

**Figure 2:**
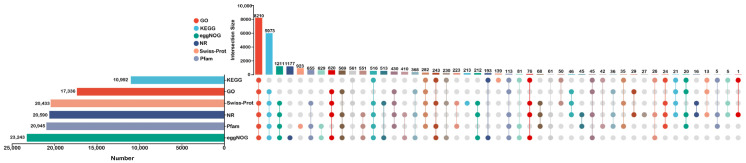
Annotation of unigenes in six public protein databases (NR, Pfam, Swiss-Prot, GO, eggnog and KEGG). The bar chart on the left represents the total number of unigenes annotated in the corresponding database, the lighted circle on the lower right indicates that unigenes have been annotated to the corresponding database; the connecting line indicates unigenes annotated simultaneously, and the number above indicates the number of unigenes annotated in the databases corresponding to the connected lighted circle.

Of the total unigenes, 47,169 were successfully annotated in at least one of the six publicly available protein databases. The annotations were distributed as follows: 17,336 unigenes (36.75%) were annotated in the Gene Ontology (GO) database, 10,992 in the KEGG database, 23,243 in the eggnog database, 20,590 in the NCBI protein non-redundant (NR) database, 20,433 in the Swiss-Prot database and 20,945 in the Pfam database (Table 3). Notably, 8210 unigenes displayed significant matches across all six databases, reflecting comprehensive annotation coverage. In addition, the number of unigenes that were individually annotated into these databases differed, such as 1177 in COG and 0 in GO (Figure 2).

### 2.4. Expression Patterns of Key Genes and Potential Genes Manipulating Phenolic Acid Biosynthesis in S. miltiorrhiza

**Figure 3:**
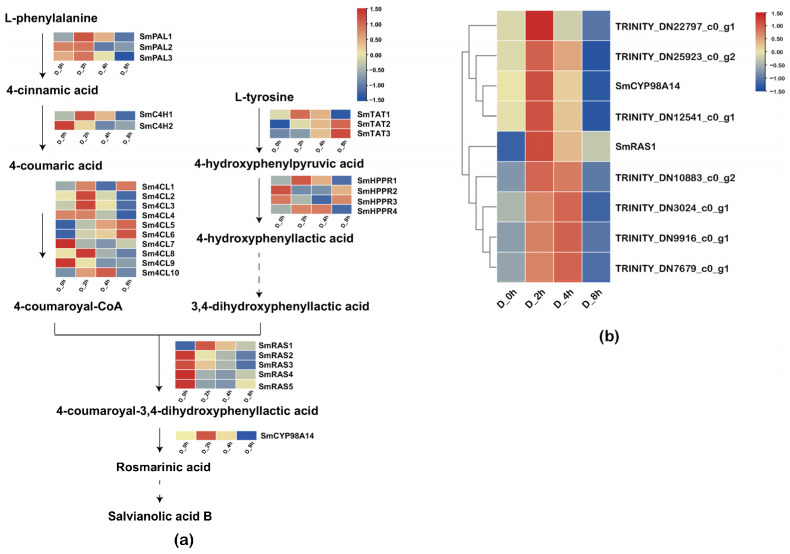
Expression profile of selected unigenes. (**a**) Expression of key enzyme genes in the biosynthetic pathway of phenolic acids. The expression levels of biosynthetic genes at different times of drought treatment were presented in a heatmap using line normalization criteria. FPKM values of unigenes were transformed by log2. The redder the color, the higher the expression level, and the bluer the color, the lower the expression level. (**b**) Correlation expression profile of selected laccases with SmRAS1 and SmCYP98A14. After FPKM normalization, 7 of the 50 unigenes annotated as LAC were found to have similar expression trends to SmRAS1 and SmCYP98A14.

The expression patterns of key genes involved in the manipulation of the phenolic acid biosynthesis pathway were investigated. Specifically, unigenes encoding enzymes, such as SmPAL1, SmC4H1, Sm4CL1, SmTAT1, SmHPPR1, SmRAS1 and SmCYP98A14, were analyzed. It was found that the expression levels of these genes were strongly induced and reached to peak at 2h. This expression pattern closely mirrored the accumulation pattern of phenolic acids (Figure 3a). The specific steps from RA to SalB have not been elucidated, and it has been speculated that laccase may play an important role in this process. Therefore, the expression pattern of laccase after drought treatment was analyzed. A total of 50 unigenes were screened and annotated as LACs in the transcriptome (Table S1), and the expression correlation analysis revealed that 7 unigenes showed similar expression patterns with SmRAS1 and SmCYP98A14 (Figure 3b). Namely, TRINITY_DN22797_c0_g1, TRINITY_DN25923_c0_g2 and TRINITY_DN12541_c0_g1 were highly correlated with SmCYP98A14, while TRINITY_DN10883_c0_g2, TRINITY_DN3024_c0_g1, TRINITY_DN9916_c0_g1 and TRINITY_DN7679_c0_g1 were highly correlated with SmRAS1, indicating that these candidate LAC genes may be involved in phenolic acid biosynthesis, which deserved further investigation.

### 2.5. Analysis of DEGs under Drought Stress

**Figure 4:**
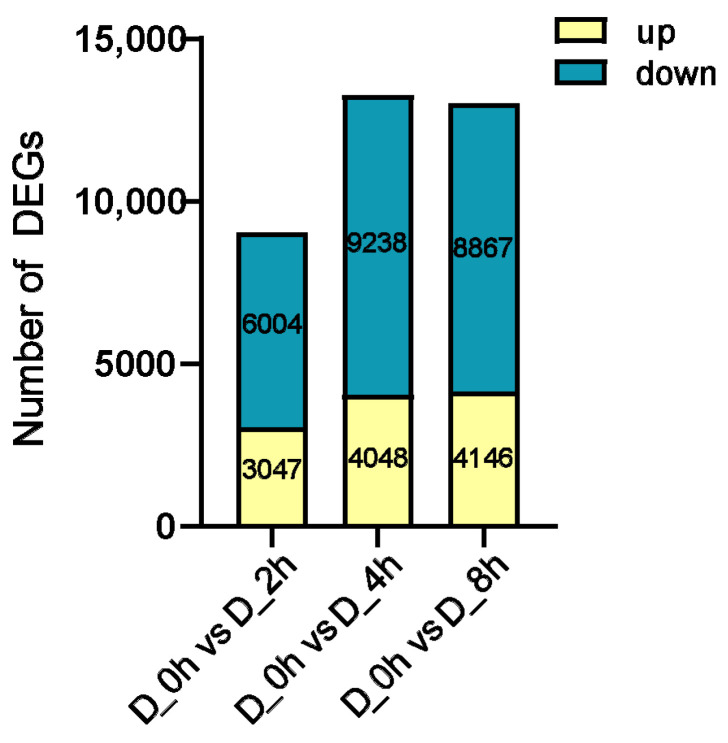
DEGs in different drought conditions. The gene expression levels of genes under drought treatment at 2 h (D_2h), 4 h (D_4h) and 8 h (D_8h) compared with the control (D_0h), respectively. “Up” meant that expression levels were significantly up-regulated compared with the control, and “down” represented down-regulation after drought treatment when compared with the control.

To investigate the impact of drought stress on *S. miltiorrhiza* at different time points, differential expressed genes (DEGs) were identified. Compared to the control group, a total of 9051 unigenes exhibited differential expression in response to drought stress at 2h, with 3047 up-regulated genes and 6004 down-regulated genes. Similarly, at 4h, 13,286 unigenes showed differential expression, with 4048 up-regulated genes and 9238 down-regulated genes. Drought stress at 8h resulted in the differential expression of 13,013 unigenes, with 4146 up-regulated genes and 8867 down-regulated genes. Notably, the highest number of DEGs (13,286) appeared in the drought-0h-vs.-drought-4h group. However, the largest number of up-regulated genes (4146) was found in the drought-0h versus drought-8h comparison (Figure 4).

### 2.6. Functional Enrichment Analysis of DEGs

**Figure 5:**
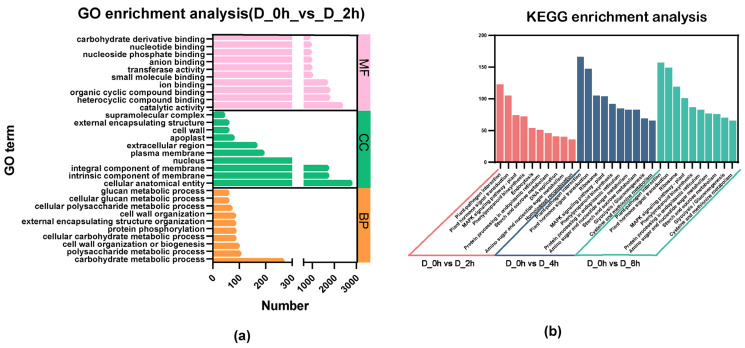
Functional enrichment analysis of DGEs. (**a**) GO enrichment analysis of DEGs in D_0h vs. D_2h. The ordinate indicates the GO term, and the abscissa indicates the number of enrichments. The top 10 terms with the largest number from MF (molecular function), BP (biological process) and CC (cellular component), respectively, were selected for display. (**b**) KEGG enrichment analysis of DEGs under drought treatment. Red, D_0h vs. D_2h; blue, D_0h vs. D_4h; green, D_0h vs. D_8h.

To explore the function of the selected DEGs, the GO database was used to perform significant enrichment analysis (Figure 5a; Figure S1). GO terms enriched by the DEGs of drought 2h and control were analyzed, among which carbohydrate metabolic process enriched in BP (biological process) was the most, and the cellular anatomical entity was enriched in the largest number in CC (cellular component). For MF (molecular function), catalytic activity owned the most number. GO terms involved in DEGs between the drought-4h treatment and the control, which were mainly concentrated in MF, and the cellular anatomical entity (CC, 4647) was the most enriched. GO terms involved in DEGs between 8 h drought treatment and control were mainly enriched in MF, and the most enriched was cellular anatomical entity (CC, 4467).

To explore the biological functions and metabolic pathways of DEGs, KEGG enrichment analysis was applied (Figure 5b). DEGs were involved in 128 metabolic pathways after 2h of drought, 131 metabolic pathways after 4 h of drought and 130 metabolic pathways after 8h of drought, among which the top 10 metabolic pathways were all phenylpropanoid biosynthesis, plant hormone signal transduction, MAPK signaling pathway in plants, plant–pathogen interaction and diterpenoid biosynthesis.

### 2.7. DEGs of Transcription Factors (TFs) under Drought Stress

**Figure 6:**
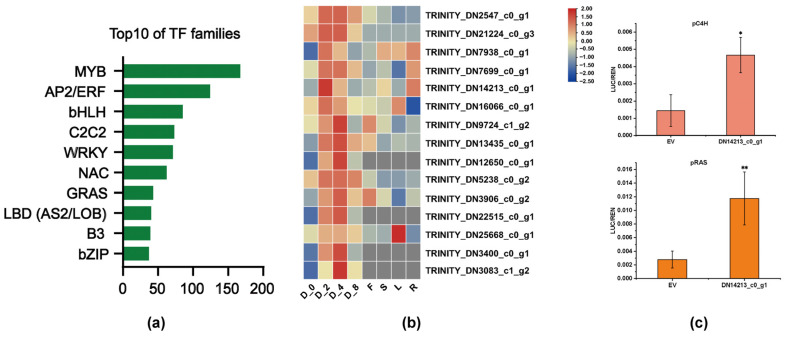
Analysis of DEGs annotated as TFs. (**a**) The top 10 TF families with more than 35 members were listed. (**b**) Expression profile of 15 TFs in different drought treatments and different tissues in *S. miltiorrhiza*. D_0h, D_2h, D_4h and D_8h represented drought treatment at 0h, 2h, 4h and 8h, respectively. F, flower; S, stem; L, leaf; R, root. FPKM values of unigenes were transformed by log2. The redder the color, the higher the expression level, and the bluer the color, the lower the expression level. (**c**) Dual-luc analysis showing that DN14213_c0_g1 activated SmC4H and SmRAS. The values were calculated by the LUC/REN ratio. Error bars show ± SD (*n* = 3), * *p* < 0.05, ** *p* < 0.01.

Using the PlantTFDB 4.0 database, a total of 1028 TFs were identified and categorized into 20 families. The top 10 TF families were determined based on having more than 35 members, and they included MYB, AP2/ERF, bHLH, C2C2, WRKY, NAC, GRAS, LBD (AS2/LOB), B3 and bZIP (Figure 6a). To investigate the potential TFs involved in regulating the accumulation of phenolic acids under drought stress, 15 differentially expressed TFs were screened. These TFs showed significant up-regulation at 2 and 4h, but no significant difference was observed after 8h (Table 4). Given that phenolic acids, such as RA and SalB, are predominantly found in the roots, the tissue expression profiles of these TFs were analyzed to identify potential candidates involved in phenolic acid biosynthesis (Figure 6b). By examining the tissue-specific expression patterns of the candidate TFs, we aimed to uncover key genes that may play a role in the biosynthesis of phenolic acids. TRINITY_DN7699_c0_g1 (AP2/ERF), TRINITY_DN14213_c0_g1 (AP2/ERF), TRINITY_DN7938_c0_g1 (LBD), TRINITY_DN3906_c0_g2 (C3H) and TRINITY_DN2547_c0_g1 (C2C2) displayed higher accumulation in roots than in other tissues. Meanwhile, the analysis revealed that TRINITY_DN14213_c0_g1 (AP2/ERF) was annotated as DREB in the Swiss-Prot database, and we conducted the Dual-luciferase (Dual-luc) assay (Figure 6c). The LUC/REN value showed that TRINITY_DN14213_c0_g1 (AP2/ERF) significantly transactivated the expression of C4H and RAS, suggesting its role in the regulation of phenolic acid biosynthesis.

### 2.8. Candidate Genes Involved in ABA Pathway Responding to Drought Stress

**Figure 7:**
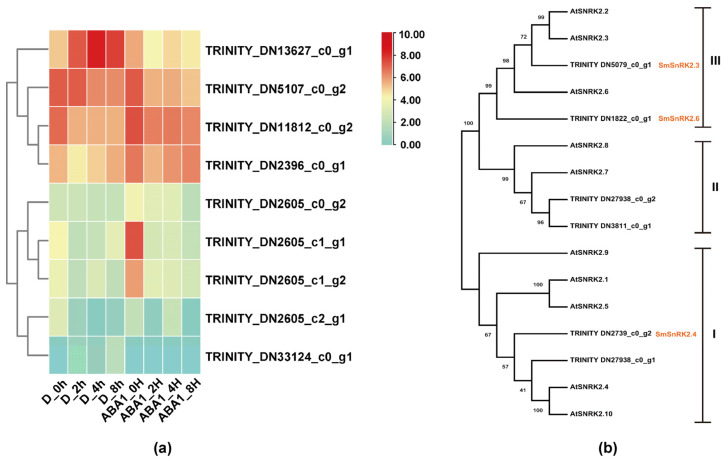
Analysis of ABA-dependent signaling pathway genes under drought stress. (**a**) Correlation analysis of 9 PYLs in drought and ABA transcriptome. D_0h, D_2h, D_4h and D_8h represented drought treatment at 0, 2, 4 and 8h, respectively. ABA1_0h, ABA1_2h, ABA1_4h and ABA1_8h represented ABA treatment of 50 μmol/L at 0, 2, 4 and 8h, respectively. FPKM values of unigenes were transformed by log2, and row cluster analysis was used. (**b**) Phylogenetic tree of 6 unigenes annotated SmSnRK2s with SnRK2 members of *A. thaliana*. The phylogenetic tree was generated using the neighbor-joining method by MEGA11 software. The numerals next to the branch nodes indicate bootstrap values from 1000 replications. AtSnRKs were downloaded from the Arabidopsis database: AtSnRK2.1 (AT5G08590), AtSnRK2.2 (AT3G50500), AtSnRK2.3 (AT5G66880), AtSnRK2.4 (AT1G10940), AtSnRK2.5 (AT5G63650), AtSnRK2.6 (AT4G33950), AtSnRK2.7 (AT4G40010), AtSnRK2.8 (AT1G78290), AtSnRK2.9 (AT2G23030) and AtSnRK2.10 (AT1G60940).

Drought stress can induce the increase in ABA, and the increasing ABA can improve the drought tolerance of plants. To investigate the ABA-dependent genes, the expression patterns of the core components of ABA signaling perception and transmission, such as PYL/PYR/RCAR, PP2C and SnRK2, were investigated. According to the comprehensive annotation of six databases, 11 unigenes were annotated as ABA receptors (PYL/PYR/RCAR) in the drought transcriptome, of which 9 unigenes were also annotated as ABA receptors (Table S2). Analysis of expression patterns in drought and ABA databases showed that TRINITY_DN13627_c0_g1, TRINITY_DN5107_c0_g2, TRINITY_DN11812_c0_g2 and TRINITY_DN2396_c0_g1 significantly responded to both drought and ABA (Figure 7a). Nine unigenes were screened to be annotated as SnRK2 family members. After the removal of the three short sequences, the phylogenetic tree of SnRK2 with 10 Arabidopsis thaliana SnRK2s showed that TRINITY_DN5079_c0_g1 (SmSnRK2.3) and TRINITY_DN1822_c0_g1 (SmSnRK2.6) belonged to SnRKIII subfamily, TRINITY_DN27938_c0_g2 and TRINITY_DN3811_c0_g1 belonged to SnRKII subfamily, which were activated by ABA (Figure 7b).

### 2.9. Expression Analysis of Selected DEGs

**Figure 8:**
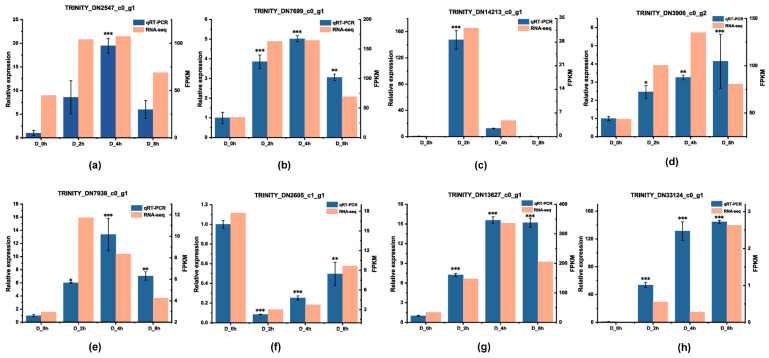
qRT-PCR and RNA-seq data of selected unigenes. (**a**–**h**) qRT-PCR and RNA-seq data of the unigenes which played important role in the biosynthesis of phenolic acids or regulation of drought stress mentioned in this study. The qRT-PCR data are shown in a blue bar chart, with a relative expression on the left Y-axis; error bars represent ± SD of triplicates for qRT-PCR, and an asterisk denotes statistically significant differences with D_0h. D_0h, drought for 0h (control); D_2h, drought for 2h; D_4h, drought for 4h; D_8h, drought for 8h. * *p* < 0.05; ** *p* < 0.01; *** *p* < 0.001. The corresponding data for selected unigenes in transcriptome data shown as RNA-seq are in the orange bar plots chart with FPKM values on the right Y-axis, and FPKM values of unigenes were transformed by log2.

In order to certify the reliability of high-throughput transcriptome sequencing and guarantee the correctness of our analysis, eight unigenes that may play an important role in phenolic acid biosynthesis under drought stress were selected for qRT-PCR analysis. Expression pattern of selected unigenes including TRINITY_DN2547_c0_g1 (C2C2), TRINITY_DN7699_c0_g1 (AP2/ERF), TRINITY_DN14213_c0_g1 (AP2/ERF), TRINITY_DN3906_c0_g2 (C3H), TRINITY_DN7938_c0_g1 (LBD), TRINITY_DN2605_c1_g1 (PYL), TRINITY_DN13627_c0_g1 (PYL) and TRINITY_DN33124_c0_g1 (PYL) displayed similar expression trends as RNA-seq, suggesting high data accuracy of the transcriptome sequencing (Figure 8).

## 3. Discussion

Plants inevitably face a series of biotic and abiotic stresses during their growth. When stress occurs, some complex physiological responses occur in plants to cope with adversity. With the intensification of global warming, drought has gradually become one of the abiotic stresses showing the greatest impact on plant growth and accumulation of distinctive substances. Water limitations have reduced the yield and quality of the crops. Maize (*Zea mays* L.) and wheat (*Triticum aestivum* L.) changed their phenotypes (leaf area, the number of grains per year) with drought conditions, resulting in a reduction in yields [24,25]. And drought could affect the accumulation of secondary metabolites in a wide variety of plant species [19,20,22]. As an important medicinal plant, *S. miltiorrhiza* produces tanshinones and phenolic acids as the main active substances [26,27]. Their production and accumulation are affected by many factors. Exogenous application of ABA, MeJA, yeast extract and Ag^+^ can stimulate the accumulation of phenolic acids and tanshinones [10,11,28]. In the present study, one-month-old *S. miltiorrhiza* seedlings were subjected to different drought durations, and total phenolic acids increased first and then decreased; the regulatory mechanism of drought on phenolic acids biosynthesis deserves further exploration.

DEGs under different drought time processing were screened. The number of differentially up-regulated genes in D_2h, D_4h and D_8h groups was 3047, 4048 and 4146, respectively, showing a gradual increase trend, suggesting that with the extension of drought stress, plants have activated a series of complex regulatory mechanisms to up-regulate the expression of related stress protection genes to adapt to or resist drought stress. TFs directly regulate the expression of downstream genes via the interaction with specific cis-elements in the promoter region, making plants adapt to drought stress or enhance plant drought tolerance. For example, *SiMYB75* confers sesame (*Sesamum indicum* L.) drought stress tolerance by promoting stomatal closure to reduce water loss and up-regulating the expression levels of various stress-marker genes in the ABA-dependent pathways [29]. Transcriptome analysis showed that the drought-induced *TaMYB31*, when ectopically expressed in *A*. *thaliana*, increased drought resistance and up-regulated some wax biosynthesis genes and drought response genes in wheat [30]. In order to explore the TFs that regulate phenolic acid biosynthesis under drought stress, we selected 15 candidate TFs based on the results of HPLC and the expression pattern analysis of key enzyme genes in the phenolic acid biosynthesis pathway. Among them, TRINITY_DN14213_c0_g1 was annotated as DREB, which displays homology with the A-1 subfamily of *AtDREB*s. DREB mainly modulates gene regulation under drought, salt and cold stress in ABA-independent pathways, and most of the A-1 subfamily responded to cold stress [31]. *AtDREB1C* (AT4G25470.1), *AtDREB1B* (AT4G25490.1) and *AtDREB1A* (AT4G25480.1) were reported to respond to cold [32,33,34]. Recent studies displayed that overexpression of *AtDREB1A* significantly increased tolerance to cold and drought in *Arabidopsis* and the capacity of drought tolerance in potatoes, and overexpression of *AtDREB1B* improved the drought resistance in *S. miltiorrhiza*. Moreover, overexpression of soybean *DREB1* increased the drought tolerance of transgenic wheat in the field [35], and overexpression of *DREB1A* from *Oryza sativa* L. increased the tolerance of transgenic *Arabidopsis* to drought, high salt and cold stress [36]. Dual-luc results showed that TRINITY_DN14213_c0_g1 significantly induced the expression of *C4H* and *RAS* with LUC/REN value higher than the control. TFs such as *SmPAP1* and *SmbZIP1* have been confirmed to play a positive role in phenolic acid accumulation by targeting *C4H* [8,16], while *SmERF115* directly modulates the expression of *RAS* [14], suggesting that TRINITY_DN14213_c0_g1 may play a vital role in phenolic acid biosynthesis.

KEGG enrichment analysis was applied to investigate the metabolic pathways mainly involved in DEGs under different drought times. Phenolic acid components belong to phenylpropanoid biosynthesis, and the significant enrichment of DEGs in this metabolic pathway may lead to increased phenolic acids. The top 10 significant metabolisms in D_2h, D_4h and D_8h groups all included phenylpropanoid biosynthesis, plant hormone signal transduction, MAPK signaling pathway in plants, plant–pathogen interaction, diterpenoid biosynthesis, etc. Under drought stress, signals are transduced through a variety of signaling pathways, involving many second messengers, plant hormones and TFs. Signaling pathways in plant responses to drought stress are mainly ABA-dependent and ABA-independent signal transduction pathways, which convert the initial drought signal into cellular responses, of which the ABA-dependent pathway is the core of drought stress and osmotic stress in plants [18,37]. Three components, PYR/PYL/RCAR, PP2C and SnRK2, are now well known as core components in ABA sensing and signaling [38]. Therefore, we investigated the core components of the ABA-dependent pathway to explore the analytical mechanism of ABA signaling in response to drought stress. A total of 11 unigenes were annotated as PYL/PYR/RCAR, of which 9 could be found in the ABA transcriptome (Table S2). The phylogenetic tree with 14 AtPYLs showed TRINITY_DN13627_c0_g1, TRINI-TY_DN5107_c0_g2 and TRINITY_DN2396_c0_g1 had high homology, and AtPYL7, AtPYL8, AtPYL9 and AtPYL10, which belong to class I, and TRINITY_DN11812_c0_g2 had high homology with AtPYL1 and AtPYR1, which belonged to class III (Figure S2c). The SnRK2 family has a total of 10 members in *Arabidopsis* and is divided into three subfamilies, of which subfamily I is ABA-independent and mainly regulated by osmotic stress, subfamily III is ABA-dependent as one of the three core components, and subfamily II is less affected by ABA [39]. There are three members of the SnRK2 family in *S. miltiorrhiza* in NCBI, SmSnRK2.3 and SmSnRK2.6 belong to subfamily III and SmSnRK2.4 belong to subfamily I, which are named TRINITY_DN5079_c0_g1, TRINITY_DN1822_c0_g1 and TRINITY_DN2739_c0_g2 in drought transcriptome, respectively. A total of nine unigenes annotated as SnRK2 were screened. In order to explore the SnRK2 family and their classification, the phylogenetic tree of 9 unigenes and 10 SnRK2s in *Arabidopsis* was analyzed, and three unigenes were removed due to their short sequences. Based on the phylogenetic tree, SmSnRK2.3 and SmSnRK2.6 were clustered with AtSnRK2.2, AtSnRK2.3 and AtSnRK2.6 in *Arabidopsis*, which was consistent with the previous study. TRINITY_DN27938_c0_g2 and TRINITY_DN3811_c0_g1 were closely related to AtSnRK2.8 and AtSnRK2.7 of the second subfamily, indicating that these two may be new members of the second subfamily of *S. miltiorrhiza* SnRK2. TRINITY_DN27938_c0_g1 was closely related to SmSnRK2.4, AtSnRK2.1, AtSnRK2.5, AtSnRK2.4, AtSnRK2.10 and AtSnRK2.9, which might be a new member of SnRK2 subfamily I. In the early stage of drought stress response, before the accumulation of endogenous ABA, the response to drought stress depends on ABA-independent pathway, and the early stress signal will increase ABA synthesis by activating the expression of 9-cis-epoxycartenoid dioxygenase 3 (NCED3) [40]. We analyzed the expression of *SmNCED3* under different drought time treatments and found that the expression of *SmNCED3* was continuously induced under drought stress, which was significantly up-regulated at 2h of drought stress, reaching 8-fold of the control. TRINITY_DN2739_c0_g2 was annotated as SmSnRK2.4, a SnRK2 class I protein kinase, and its expression was the highest among all annotated members of the SnRK2 family, indicating that the ABA-independent regulatory system may play an important role in response to drought stress as the ABA-dependent system. The transcription factors phosphorylated by SnRK2s in subfamily II and subfamily III of the ABA-dependent regulatory pathway contain AREBs/ABFs, which regulate ABA-responsive genes by recognizing ABA-response element (ABRE) motifs and cause stomata closure, thus improving drought tolerance of plants [41]. Eight unigenes annotated as ABFs were found in the drought transcriptome, among which TRINITY_DN4514_c0_g1 and TRINITY_DN817_c0_g1 were significantly induced under drought stress, suggesting their potential role in response to drought stress in ABA-dependent regulatory pathways.

## 4. Materials and Methods

### 4.1. Plant Material and Stress Treatment

Aseptic *S. miltiorrhiza* plants were grown in the greenhouse of our lab in Zhejiang Chinese Medical University at 25 °C with 16h/8h (light/dark) periods in Murashige and Skoog (MS) basal medium containing 3% sugar and 0.8% agar at the pH of 5.8. One-month-old plants in consistent growth were selected and removed from the culture medium, washed off, dried out and placed in a light incubator (RXZ-500C, Ningbo Jiangnan Instrument, Ningbo, China) at 2000 lx, 25 °C under natural drought treatment. Whole plants were collected after treatment for 0, 2, 4 and 8h, respectively (Figure S3), for HPLC determination of phenolic acids and RNA extraction.

### 4.2. Analysis of Phenolic Acids

Plant samples were dried at 55 °C and then ground. About 0.1 mg of plant powder was weighed into a 2 mL EP tube and then subjected to ultrasound with 2 mL of 80% methanol for 1 h (KunshanKQ-500B, Kunshan UL transonic instruments, Kunshan, China). The solution was centrifuged with 6500× *g* for 5 min to obtain supernatant (Eppendorf, Hauppauge, NY, USA) and then filtered with 0.22 μm filter membrane. Agilent 1260 Infinity II was used for the HPLC analysis detector equipped with a reversed-phase C18 symmetry column (Agilent, Santa Clara, CA, USA). Detection parameters of phenolic acid are as follows: mobile phase: water/acetonitrile (7:3, *v*/*v*) with pH adjusted to 2.03, flow speed was 1 mL/min and detection wavelength was set to 281 nm. Individual standards were applied to quantify the content of RA and SalB, and the sum of these two compounds amounted to total phenolic acids.

### 4.3. RNA Isolation and Qualification

Total RNA was extracted from plant samples. Nanodrop2000 (Thermo Fisher Scientific, Waltham, MA, USA) was used to detect the concentration and purity of the extracted RNA; agarose gel electrophoresis was used to detect the RNA integrity; and Agilent5300 (Agilent, USA) was used to determine the RIN value.

### 4.4. Library Preparation for Transcriptome Sequencing

Transcriptome library was constructed with 1 μg of total RNA with concentration ≥ 30 ng/μL, RIN > 6.5 and OD260/280 between 1.8 and 2.2. Messenger RNA was isolated using the polyA selection method with oligo (dT) beads. Subsequently, the isolated mRNA was fragmented, and then double-stranded cDNA was synthesized with random hexamer primers. Synthesized cDNA underwent end-repair, phosphorylation and the addition of an “A” base. Libraries were size-selected for cDNA fragments of approximately 300 bp using 2% Low Range Ultra Agarose. Subsequently, PCR amplification was performed using Phusion DNA polymerase (NEB) for 15 PCR cycles (T100 Thermal Cycler, BIO-RAD, Hercules, CA, USA). The quantification of the libraries was carried out using the TBS380 system. The NovaSeq 6000 sequencer (Illumina, San Diego, CA, USA) was used to perform sequencing, with a read length of 2 × 150 bp [42].

### 4.5. Data Filtering and De Novo Assembly

In order to obtain sequencing data in high quality, the raw data of all sequenced samples should be purified and filtered to retain high-quality reads before assembly. The joint sequence was removed from the original sequencing data and also the low-quality sequence (5′ end < 20; 3′ end < 3); reads containing N (fuzzy base), adapters and sequences shorter than 30 bp, during the quality trimming process, underwent a filtration step to retain only high-quality clean reads. These retained reads were then subjected to a de novo assembly using the Trinity program.

### 4.6. Unique Sequence Functional Annotation and Classification

Diamond (https://github.com/bbuchfink/diamond, accessed on 25 August 2022) and HMMER (ftp://selab.janelia.org/pub/software/hmmer3/3.0/hmmer-3.0.tar.gz, accessed on 25 August 2022) were used to search for assembled transcripts in NR, COG, KEGG, Pfam, Swiss-Prot and GO databases. The alignment was performed using BLASTX, aiming to identify proteins with the highest sequence similarity to the transcripts and retrieve their functional annotations, and a stringent cut-off E-value less than 1.0 × 10^−5^ was set.

### 4.7. Quantification of Gene Expression Levels and Differential Expression Analysis

RSEM was used to quantify gene abundances. Essentially, differential expression analysis was performed using DESeq2 (http://bioconductor.org/packages/stats/bioc/DESeq2/, accessed on 25 August 2022) or DEGseq (https://www.rdocumentation.org/packages/DEGseq/versions/1.26.0, accessed on 25 August 2022). Unigenes with |log2FC| ≥ 1 and FDR ≤ 0.05 (DESeq2) or FDR ≤ 0.001 (DEGseq) were considered to be DEGs. Furthermore, functional enrichment analyses were conducted to identify DEGs significantly enriched in specific GO terms and metabolic pathways, which were performed with a significance threshold of *p*-adjust ≤ 0.05 when compared to the whole transcriptome background.

### 4.8. Dual-Luc Assay

All the pGREEN0800-promoter (reporter) (promoters of key genes involved in phenolic acid biosynthesis pathway, such as *SmPAL1*, *SmTAT1*, *Sm4CL1*, *SmC4H1*, *SmHPPR1*, *SmRAS1* and *SmCYP98A14*) vectors were transformed into Agrobacterium GV3101 with the helper plasmid pSoup-P19. The full-length cDNA sequence of DN14213_c0_g1 was cloned into pHB-YFP to form the effector, together with pHB-YFP as the negative control, and then moved into GV3101. GV3101 harboring the effector or reporter was applied to infiltrate the back leaves of *Nicotiana benthamiana*. After 24 h dark/24 h light, the infected area was sampled for detection using the Dual-Luciferase Reporter Assay System (Promega, Madison, WI, USA). The LUC/REN ratio indicated the ability of DN14213_c0_g1 to bind to and activate gene promoters.

### 4.9. Analysis of Gene Expression Level

Samples, drought treated for 0 h, 2 h, 4 h and 8 h, were frozen in liquid nitrogen, and total RNA was extracted using the Tiangen plant total RNA extraction kit. The qRT-PCR experiments were carried out using Thermo Fisher quantitative master mix on the Applied Biosystem Step One Real-Time PCR System (Applied Biosystems, Foster City, CA, USA), with *SmActin* as the internal control. The relative expression level of selected genes was calculated using the 2^–ΔΔCT^ method. The experiments were conducted three times.

## 5. Conclusions

Drought affects the accumulation of secondary metabolites in *S. miltiorrhiza*, but the specific mechanism is still unclear. Comprehensive transcriptome under different drought treatments in *S. miltiorrhiza* was conducted in our study. Unigene annotated as DREB (TRINITY_DN14213_c0_g1) was screened, which is closely related to the A-1 sub-family and promoted *SmC4H* and *SmRAS* expression, suggesting involvement in the biosynthesis of phenolic acids. The ABA pathway plays an important role in plant resistance to drought; four PYL members were significantly responsive to both ABA and drought, and three potential SnRK2 family members were also analyzed. This drought transcriptome will provide new insights into the molecular mechanism of phenolic acid biosynthesis under drought stress.

## Figures and Tables

**Table 1:** Summary of transcripts induced by different times after drought stress in *S. miltiorrhiza*.

Sample	Raw Reads	Raw Bases	Clean Reads	Clean Bases	Error Rate (%)	Q20 (%)	Q30 (%)	GC Content (%)
D_0h	52,876,386	7,984,334,286	52,499,006	7,692,810,276	0.0235	98.59	95.66	49.75
D_2h	53,994,332	8,153,144,132	53,596,350	7,812,370,016	0.0233	98.68	95.95	49.92
D_4h	50,632,334	7,645,482,434	50,209,782	7,314,924,392	0.0233	98.67	95.94	49.33
D_8h	53,097,080	8,017,659,080	52,658,780	7,610,216,278	0.0233	98.68	95.98	49.4

**Table 2:** Summary of sequence assembly results.

Type	Resource
Total number	95,836
Total base	105,260,765
Largest length (bp)	13,359
Smallest length (bp)	201
Average length (bp)	1098.34
N50 length (bp)	1738
E90N50 length (bp)	2071
Mean mapped percent (%)	86.204
GC percent (%)	43.47
TransRate score	0.34775
BUSCO score	C: 88.2% (S: 54.0%, D: 34.2%), F: 4.5%, M: 7.3%, *n*: 1440

**Table 3:** Functional annotation of unigenes in different databases.

Database	Exp_Unigene Number (Percent)
GO	17,336 (0.3675)
KEGG	10,992 (0.233)
eggNOG	23,243 (0.4928)
NR	20,590 (0.4365)
Swiss-Prot	20,433 (0.4332)
Pfam	20,945 (0.444)
Total_anno	25,936 (0.5499)
Total	47,169 (1)

**Table 4:** Fifteen TFs with screening criteria were selected.

Gene_ID	Family	D_0h vs. D_2h	D_0h vs. D_4h	D_0h vs. D_8h
TRINITY_DN2547_c0_g1	C2C2	up	up	no
TRINITY_DN21224_c0_g3	MYB	up	up	no
TRINITY_DN7938_c0_g1	LBD (AS2/LOB)	up	up	no
TRINITY_DN7699_c0_g1	AP2/ERF	up	up	no
TRINITY_DN14213_c0_g1	AP2/ERF	up	up	no
TRINITY_DN16066_c0_g1	AP2/ERF	up	up	no
TRINITY_DN9724_c1_g2	AP2/ERF	up	up	no
TRINITY_DN13435_c0_g1	WRKY	up	up	no
TRINITY_DN12650_c0_g1	WRKY	up	up	no
TRINITY_DN5238_c0_g2	B3	up	up	no
TRINITY_DN3906_c0_g2	C3H	up	up	no
TRINITY_DN22515_c0_g1	CAMTA	up	up	no
TRINITY_DN25668_c0_g1	bHLH	up	up	no
TRINITY_DN3400_c0_g1	bHLH	up	up	no
TRINITY_DN3083_c1_g2	CPP	up	up	no

## Data Availability

The data presented in this study are available in the article or Supplementary Materials.

## Supplementary Materials

The following supporting information can be downloaded at https://www.mdpi.com/article/10.3390/plants13020161/s1, Figure S1. GO functional enrichment analysis of DEGs. (a) GO enrichment analysis of DEGs in D_0h vs. D_4h. (b) GO enrichment analysis of DEGs in D_0h vs. D_8h. The ordinate indicates the GO term, and the abscissa indicates the number of enrichments. The top 10 terms with the largest number were selected for display from MF (molecular function), BP (biological process) and CC (cellular component), respectively; Figure S2. Selected unigenes with gene family in *Arabidopsis.* The phylogenetic tree was generated using the neighbor-joining method by MEGA11 software. The numerals next to the branch nodes indicate bootstrap values from 1000 replications. (a) The phylogenetic tree includes TF annotated as DREB (TRINITY_DN14213_c0_g1) and the *Arabidopsis* DREB gene family. The *Arabidopsis* DREB gene family was downloaded from the *Arabidopsis* database. (b) Phylogenetic analysis of selected unigene (TRINITY_DN14213_c0_g1) and *Arabidopsis* DREB A-1 subfamily including AT1G12610.1, AT1G63030.1, AT5G51990.1, AT4G25490.1, AT4G25470.1 and AT4G25480.1. (c) Phylogenetic analysis of 9 PYL/PYR/RCAR both annotated in drought and ABA transcriptome with the *Arabidopsis* PYL/PYR/RCAR family. AtPYLs were downloaded from the *Arabidopsis* database. AtPYLs were downloaded from the *Arabidopsis* database: *AtPYL1* (AT5G46790), *AtPYL2* (AT2G26040), *AtPYL3* (AT1G73000), *AtPYL4* (AT2G38310), *AtPYL5* (AT5G05440), *AtPYL6* (AT2G40330), *AtPYL7* (AT4G01026), *AtPYL8* (AT5G53160), *AtPYL9* (AT1G01360), *AtPYL10* (AT4G27920), *AtPYL11* (AT5G45860), *AtPYL12* (AT5G45870), *AtPYL13* (AT4G18620), *AtPYR1/RCAR11* (AT4G17870); Figure S3. Physiological state of *S. miltiorrhiza* plants at 0 h, 2 h, 4 h, 8 h of drought treatment; Table S1. 50 unigenes were annotated as laccase; Table S2. 11 unigenes were annotated as PYL/PYR/RCAR and 9 of them were annotated in both drought and ABA transcriptome databases.

## List of Abbreviations

RA, rosmarinic acid; SalB, phenolic acid B; PAL, phenylalanine ammonia-lyase; C4H, cinnamic acid-4-hydroxylase; 4CL, 4-coumaric acid-CoA ligase; TAT, tyrosine aminotransferase; HPPR, 4-hydroxyphenylpyruvate reductase; RAS, rosmarinic acid synthase; CYP98A14, cytochrome P450-dependent monooxygenase; LAC, laccase; MeJA, methyl jasmonate; SA, salicylic acid; ABA, abscisic acid; TF, transcription factor; HPLC, high-performance liquid chromatography; TS, total phenolic acids; D_0h, drought treatment for 0 h (before treatment); D_2h, drought treatment for 2 h; D_4h, drought treatment for 4 h; D_8h, drought treatment for 8h; DEG, differential expressed gene; BP, biological process; CC, cellular component; MF, molecular function; Dual-luc, dual-luciferase; NCED3, 9-cis-epoxycartenoid dioxygenase 3; ABRE, ABA-response element.
