# Supplementary material for: Transcriptome Analysis of Salvia miltiorrhiza under Drought Stress
Source: Plants (Basel). 2024 Jan 6;13(2):161. doi: 10.3390/plants13020161 (PMC10819027; doi:10.3390/plants13020161)

Figure S1. GO functional enrichment analysis of DEGs. **(a)** GO enrichment analysis of DEGs in D_0h vs D_4h. **(b)** GO enrichment analysis of DEGs in D_0h vs D_8h. The ordinate indicates the GO term, and the abscissa indicates the number of enrichment. Select the Top ten terms with the largest number were selected for display from MF (molecular_function), BP (biological_process) and CC (cellular_component), respectively.

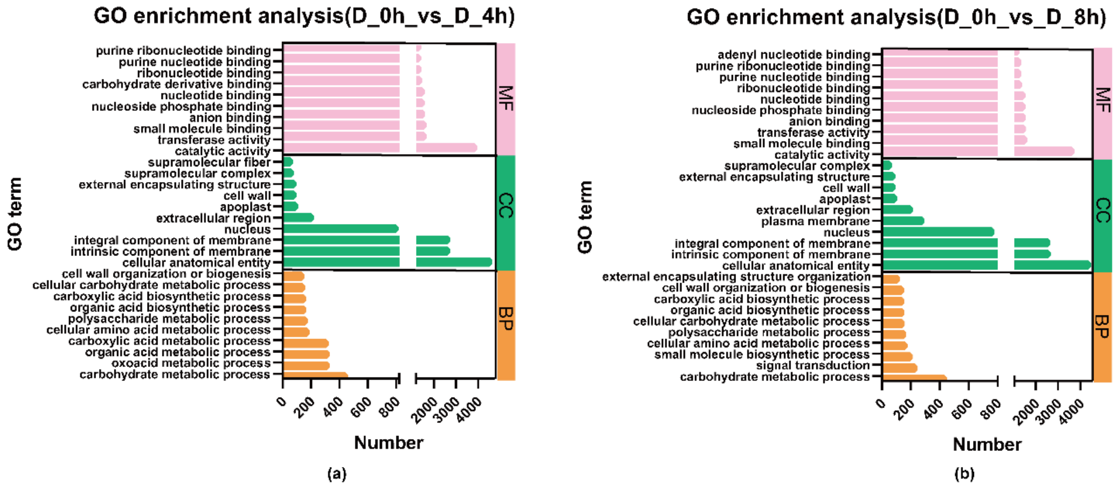

Figure S2. Selected unigenes with gene family in *Arabidopsis.* Phylogenetic tree was generated using the neighbor-joining method by MEGA11 software. The numerals next to the branch nodes indicate bootstrap values from 1000 replications. **(a)** The phylogenetic tree includes TF annotated as DREB (TRINITY_DN14213_c0_g1) and the *Arabidopsis* DREB gene family. *Arabidopsis* DREB gene family was downloaded from the *Arabidopsis* database. **(b)** Phylogenetic analysis of selected unigene (TRINITY_DN14213_c0_g1) and *Arabidopsis* DREB A-1 subfamily including AT1G12610.1, AT1G63030.1, AT5G51990.1, AT4G25490.1, AT4G25470.1, AT4G25480.1. **(c)** Phylogenetic analysis of 9 PYL/PYR/RCAR both annotated in drought and ABA transcriptome with the *Arabidopsis* PYL/PYR/RCAR family. AtPYLs were downloaded from the *Arabidopsis* database. *AtPYL1* (AT5G46790), *AtPYL2* (AT2G26040), *AtPYL3* (AT1G73000), *AtPYL4* (AT2G38310), *AtPYL5* (AT5G05440), *AtPYL6* (AT2G40330), *AtPYL7* (AT4G01026), *AtPYL8* (AT5G53160), *AtPYL9* (AT1G01360), *AtPYL10* (AT4G27920), *AtPYL11* (AT5G45860), *AtPYL12* (AT5G45870), *AtPYL13* (AT4G18620), *AtPYR1/RCAR11* (AT4G17870).

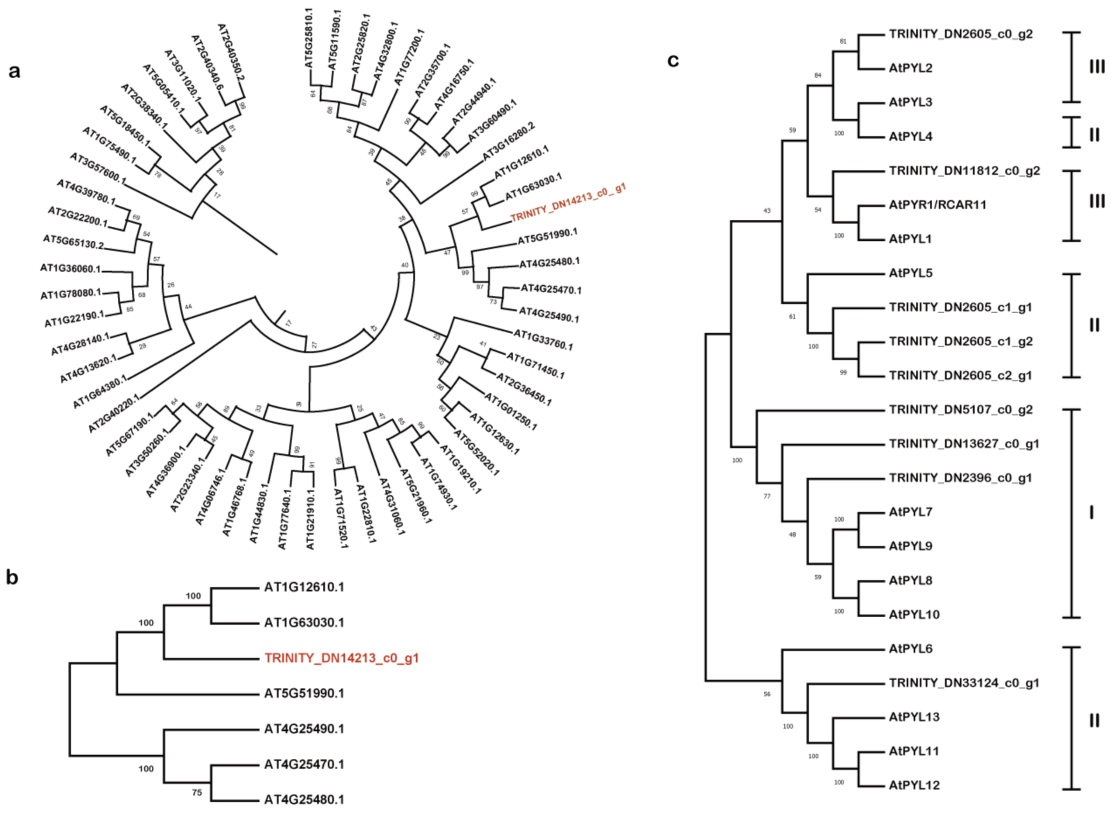

Figure S3. Physiological state of *S. miltiorrhiza* plants at 0h, 2h, 4h, 8h of drought treatment.

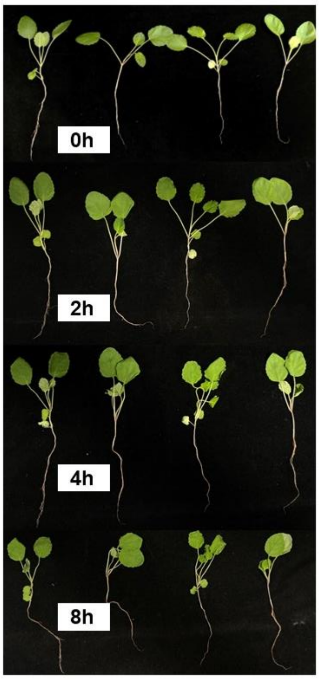

Supplement: Supplementary file 1 [file plants-13-00161-s001.zip › supplementary Figures.pdf]
